# Management of pure intracavernous chondroma: a case report with operative video and literature review

**DOI:** 10.3389/fonc.2026.1712593

**Published:** 2026-04-13

**Authors:** Alberto Acitores Cancela, Abdullah Keles, Ufuk Erginoglu, Jeffrey J. Helgager, Mustafa Kemal Baskaya

**Affiliations:** 1 Department of Neurological Surgery, School of Medicine and Public Health, University of Wisconsin, Madison, WI, United States; 2 Department of Pathology and Laboratory Medicine, School of Medicine and Public Health, University of Wisconsin, Madison, WI, United States

**Keywords:** cavernous sinus, chondroma, intracavernous chondroma, microsurgical resection, middle cranial fossa, skull base tumor, transcavernous approach

## Abstract

The cavernous sinus, often referred to as an “anatomic jewel box” due to its complex structure, presents significant challenges for neurosurgeons when performing tumor resections in this region. Tumors confined to the cavernous sinus are rare, with meningiomas and cranial nerve schwannomas being the most common. Pure intracavernous chondromas, however, are exceptionally rare, representing only a small fraction of intracranial chondromas. To date, only seven cases of pure intracavernous chondromas have been reported in the literature. Here, we report the case of a 37-year-old male who presented with a two-month history of double vision and radiological wor=90*--------up revealed a pure intracavernous sinus lesion. Gross total resection was achieved via a pterional craniotomy with a pretemporal transcavernous approach and extradural anterior clinoidectomy. The lesion was identified as a pure intracavernous sinus chondroma through a combination of radiological imaging, intraoperative observations, and histopathological analysis. At the most recent follow-up, the patient’s preoperative symptoms had been resolved, and he remained recurrence-free. We also conducted a review of the literature on pure intracavernous chondromas, examining the number of reported cases, demographics, clinical presentations, surgical management, and outcomes. The review highlighted the rarity of these tumors, the challenges in diagnosing them due to their radiological overlap with other skull base lesions, and the favorable outcomes associated with gross total resection. This case report emphasizes the importance of considering pure intracavernous chondromas in the differential diagnosis of cavernous sinus tumors and provides insights into their optimal surgical management.

## 1 Introduction

Chondromas are benign, well-circumscribed tumors composed of mature hyaline cartilage (1). They can occur as isolated lesions or as part of conditions such as Ollier’s disease and Maffucci’s syndrome. Intracranial chondromas are rare, constituting less than 0.5% of all intracranial tumors (2). These tumors typically arise in regions with bone formed through endochondral ossification, such as the sellar and petroclival areas (3). Most intracranial chondromas present in individuals during the third decade of life, with no gender predilection (2, 4, 5).

Although intracranial chondromas are rare, those confined to the cavernous sinus (CS) are even more exceptional. The CS is one of the most anatomically complex regions in the human body, often described by D. Parkinson in 1987 as a “veritable anatomic jewel box” due to its intricate structure (6). As a result, performing tumor resection in the CS remains a significant neurosurgical challenge. Tumors confined exclusively to the CS are rare and typically occur in adults, with meningiomas and cranial nerve schwannomas being the most common types (7). Descriptions of CS chondromas in the literature are limited, and pure intracavernous chondromas confined entirely to the CS are even more exceptionally rare (5, 8–10).

Intracranial chondromas are slow-growing tumors, accounting for only 0.2% of all primary intracranial tumors (11). They are usually solitary but can be associated with syndromic conditions such as Ollier’s disease and Maffucci’s syndrome (12). Several mechanisms have been proposed for their origin, including persistence of embryonic chondrocytes along skull base synchondroses (11, 13), metaplasia of meningeal fibroblasts (11, 12, 14), and reactive changes of displaced fibro-cartilaginous elements after trauma or inflammation (15).

While the literature on CS tumors is expanding, reported cases of pure intracavernous sinus chondromas remain exceedingly rare. This highlights a gap in our understanding of the optimal approach to diagnosis, surgical management, and long-term follow-up for these lesions. Given the limited number of documented cases, further exploration of the clinical presentation, imaging characteristics, and outcomes associated with CS chondromas is essential. This study aims to contribute to the literature by presenting a new case of a pure intracavernous chondroma, accompanied by review of existing cases published in the literature. By synthesizing these findings, we hope to provide insights into optimal treatment strategies and improve patient outcomes for this rare condition.

### 1.1 Case report

**Figure 1:**
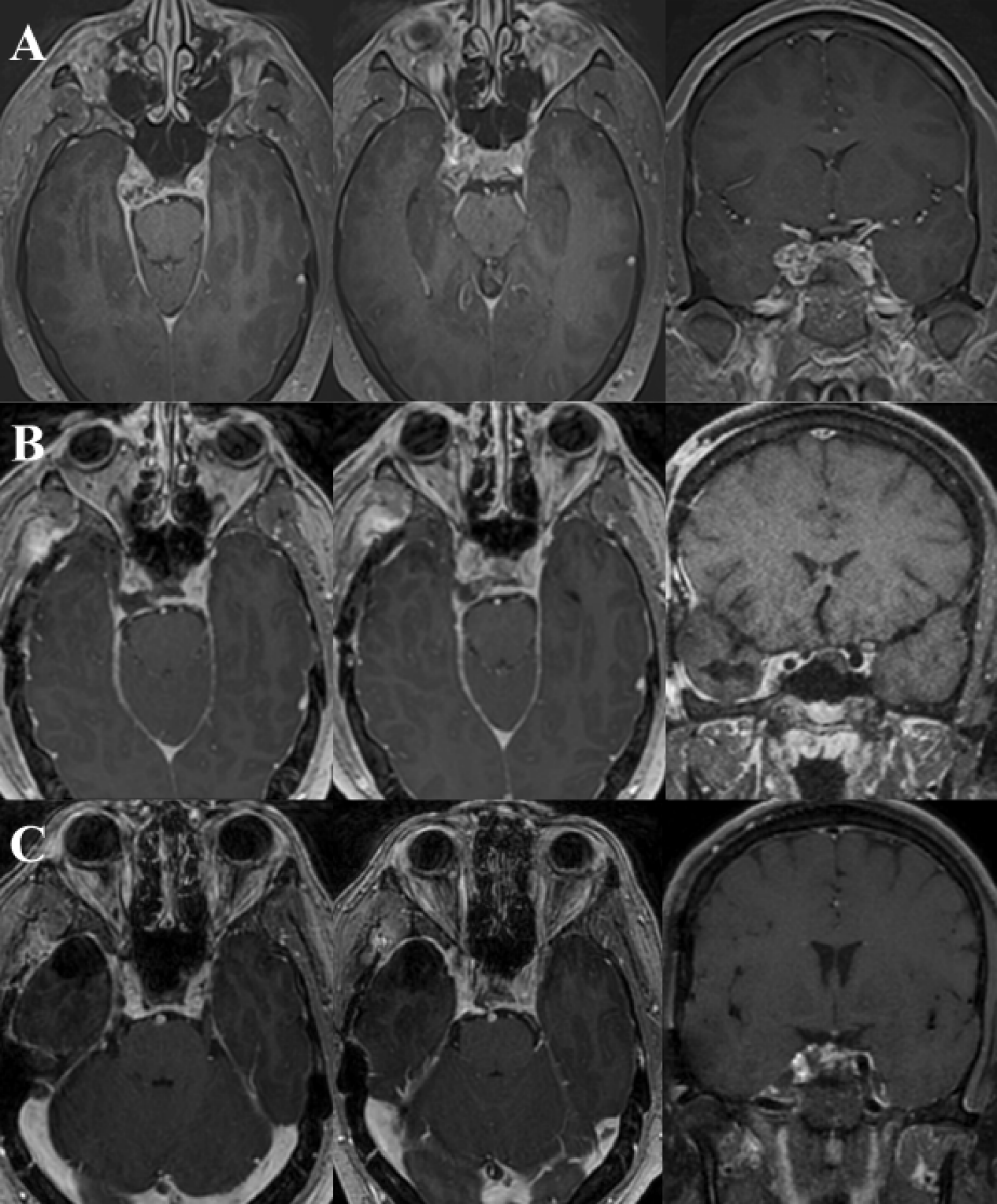
**(A)** Preoperative T1-weighted contrast-enhanced magnetic resonance image (MRI) of brain demonstrating axial and coronal views of a well-demarcated enhancing lesion within right cavernous sinus. **(B)** Postoperative axial and coronal views showing minimal residual enhancement along the right cavernous sinus. **(C)** Four-year follow-up MRI demonstrating stable appearance of the residual lesion with no evidence of progression.

A 37-year-old male presented with a two-month history of double vision without neurological motor deficits. Computed tomography (CT) revealed an intracavernous lesion. His past medical, surgical, family, and social histories were unremarkable. Magnetic resonance imaging (MRI) further confirmed a right intracavernous lesion measuring 2.5x1.5x2.0cm (**Figure 1A**). The mass exhibited thin but strong peripheral enhancement, with foci of faint T1 hyperintensity and low susceptibility on gradient echo images, suggesting calcifications. On T2-weighted images, the lesion showed peripheral low and central high signal intensity, further supporting the presence of calcified areas.

This study was approved by the hospital’s Institutional Review Board, and the patient provided consent for the procedure and the publication of anonymized medical information.

### 1.2 Surgical technique

After the induction of general anesthesia, the patient was positioned laterally, and a lumbar drain was placed to relax the temporal lobe during dissection of the cranial middle fossa. A right pterional craniotomy was performed, followed by drilling of the greater wing of the sphenoid. The dura propria was carefully elevated from the lateral wall of the CS. The superior orbital fissure was unlocked, the optic nerve was unroofed, and an extradural anterior clinoidectomy was completed.

Under microsurgical dissection, the outer dural layer overlying the medial temporal lobe was lateralized to expose the three divisions of the trigeminal nerve. The tumor, protruding between the V1 and V2 divisions, was identified. Doppler ultrasound was used to confirm the location of the intracavernous internal carotid artery (ICA). Tumor content was evacuated using a combination of sharp and blunt dissection. A mixture of bony fragments and soft, grayish tumor material was removed. Microsurgical near-total resection was achieved, with a small, calcified fragment adherent to the ICA left behind. The bone flap was replaced and secured with titanium plates and screws. Standard closure of the scalp was performed using 2–0 Vicryl for the galea and 4–0 Prolene for the skin. A video demonstration of the surgical resection is provided (**Video 1**). The postoperative course was uneventful, and the patient was discharged on postoperative day 3.

**Figure 2:**
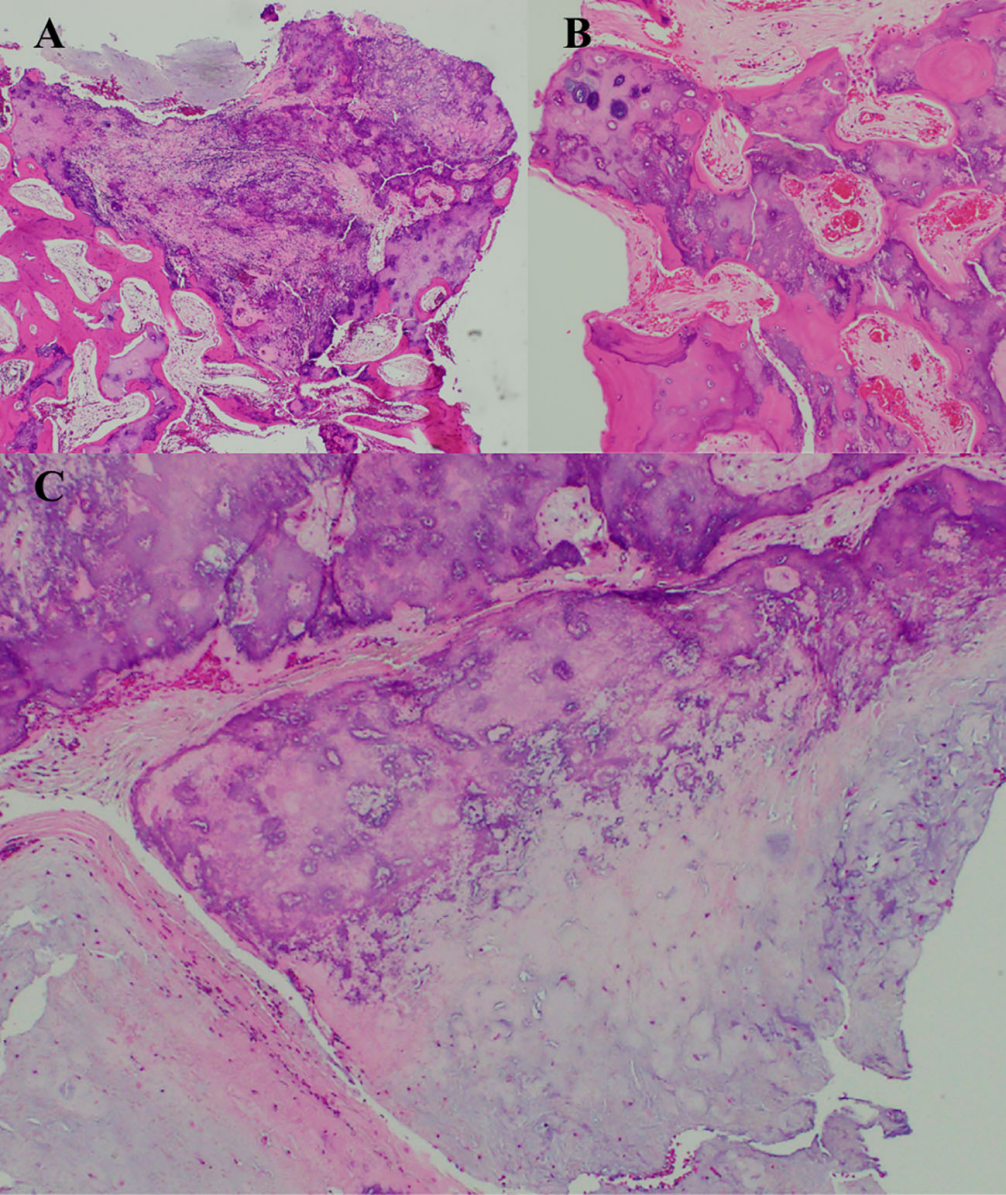
Pathology of indolent-appearing osteocartilaginous lesion with dense calcifications. **(A)** Despite fragmentation, limiting the evaluation of invasive growth pattern, the lesion appears well-circumscribed and lobulated in areas where the interface of the lesion apposing bone is evaluable. **(B)** Many areas are densely calcified with enchondral ossification. **(C)** Hyaline cartilage portions with no more than mild atypia, no significant increase in cellularity, necrosis, or mitotic activity; heavy calcification is again evident. Though difficult to classify precisely, the cytomorphologic features are most consistent with an indolent hyaline cartilage neoplasm, and in conjunction with radiographic findings favored to represent a chondroma.

Histopathological examination revealed a well-circumscribed, lobulated lesion with dense calcifications and areas of enchondral ossification. The tumor consisted predominantly of hyaline cartilage with mild atypia, but no significant increase in cellularity, necrosis, or mitotic activity. The lesion’s low cellularity and heavy calcifications made precise classification challenging, but the cytomorphologic features were most consistent with an indolent hyaline cartilage neoplasm. In conjunction with radiological findings, the tumor was favored to represent a chondroma (**Figure 2**).

At the one-month follow-up, the patient reported no further symptoms of double vision. Follow-up MRI at two months showed no significant changes in the small residual tumor fragment adherent to the intracavernous ICA (**Figure 1B**). The patient remained asymptomatic with stable neurological function. At the 4-year follow-up, MRI confirmed no growth of the residual lesion (**Figure 1C**), and the patient continues to be neurologically stable without recurrence of symptoms.

## 2 Materials and methods

**Figure 3:**
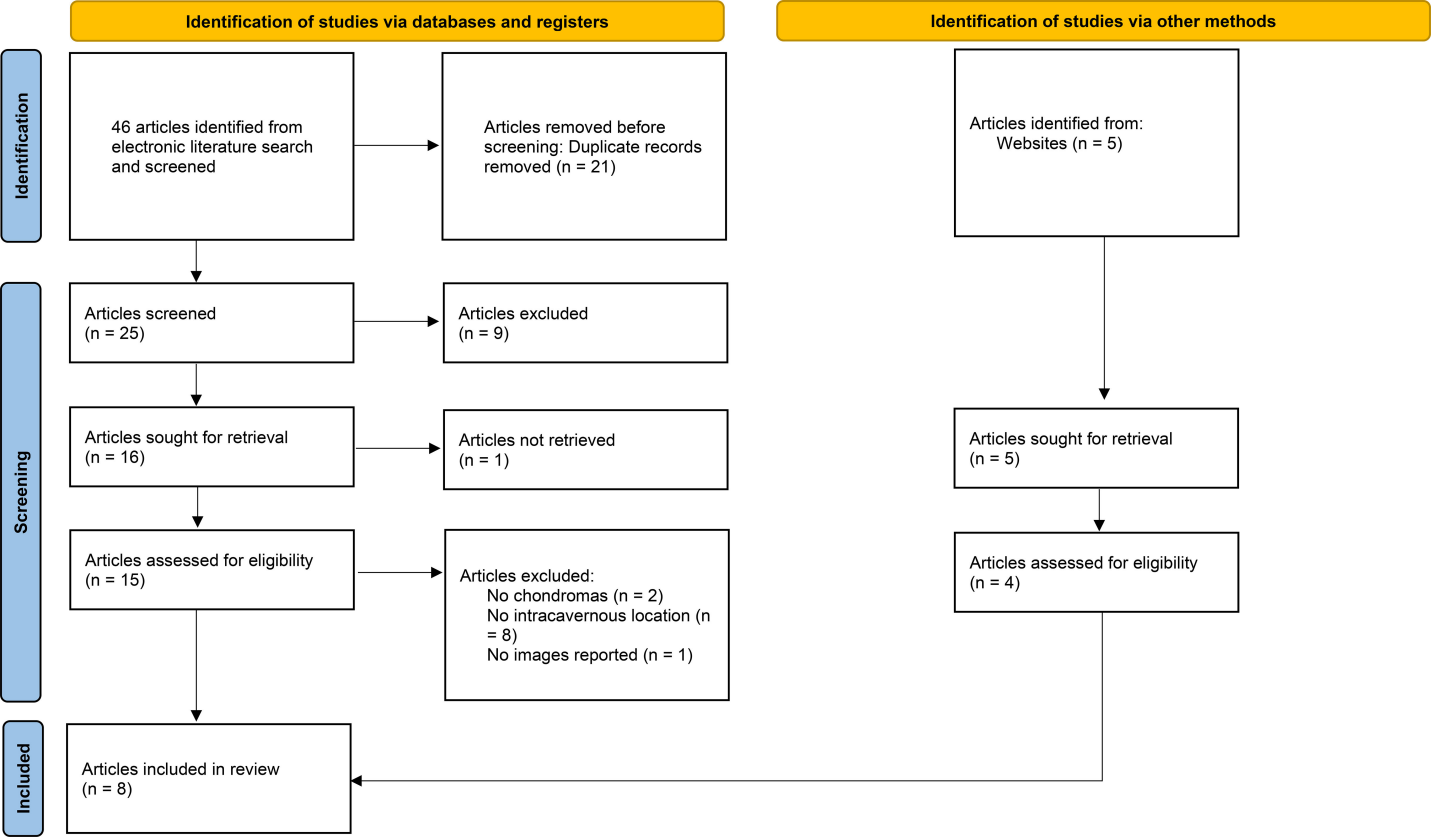
Flowchart of the literature search.

A thorough literature review was conducted to identify reports on CS chondromas using the following databases: Medline, Web of Science, ResearchGate, and Google Scholar, for papers published until October 2024. Abstracts in English, Spanish, French, Chinese, and Portuguese were included. In addition, the reference lists of selected articles were manually reviewed to identify relevant studies. All article types were considered for inclusion. Studies were eligible for inclusion if they described chondromas confined entirely to the CS. Exclusion criteria included reports of tumors extending outside the CS, or studies lacking sufficient clinical or radiological data for analysis. To manage references and exclude duplicates and non-relevant studies, Mendeley reference management software (Elsevier, Amsterdam) was utilized. The process for literature inclusion and exclusion followed the PRISMA guidelines, as outlined in the flowchart for systematic reviews (16). A total of 8 studies were included in the review after applying the inclusion and exclusion criteria (**Figure 3**).

## 3 Results

### 3.1 Literature search results

Our literature review identified eight articles reporting intracavernous chondromas. After careful evaluation, only seven unique cases were confirmed as chondromas fully confined to the CS (**Table 1**) (4, 5, 7, 8, 17–20). Notably, one patient was reported twice, first in 2016 and later in 2022 (17, 18). One additional case was excluded because review of its CT and MRI images demonstrated the tumor was not entirely intracavernous, and the authors described it as an osteochondroma (21). Another report was excluded as it was limited to an abstract describing an intracavernous chondroma causing basilar artery compression without clinical or radiological images (22).

**Table 1:** Patient characteristics and outcomes of the reviewed cases.

Case N°	Reference	Classification (regarding origin)	Year	Age	Sex	Symptoms	EOR	Surgical approach	Origin	Pathology	Adjuvant treatment	Follow-up and outcome
1	Lesoin et al. (8)	Intracavernous	1987	36	M	CN III, incomplete	STR	OZ	NS	Chondroma	NS	NS
2	Terasaka et al. (19)	Intracavernous	1997	40	F	CN III and CN VI unilateral	GTR	OZ	Right post clinoid	Mature chondroma	No	Post-operative right ophtalmoplegia during 6m, then asymptomatic.
3	Tu et al. (20)	Intracavernous	2000	25	M	H/A, diplopia, facial numbness	GTR	OZ, extradural	NS	Chondroma	Second surgery (small residual tumor posteromedial part of the left cavernous sinus. 1st surgery: FOZ extradural).	Transient VI nerve palsy.
4	Fratzoglou et al. (4)	Intracavernous, interdural.	2008	50	M	Paresis of CN VI left, corneal diminished	STR	Pterional	Between layers of cavernous sinus	Mature chondroma	NS	Hemifacial pain disappeared and the diplopia improved from the first postoperative day.
5	Huang et al. & Su et al. (17, 18)	Intracavernous	2016/2022	30	F	Hypometropia, amenorrhea, and lactation	GTR	FT + EDA	NS	Chondroma	NS	NS
6	Valdez et al. (5)	Intracavernous	2015	41	F	Left CN VI	GTR	FT	NS	Chondroma	NS	Normal abducens nerve function the day after surgery.
7	Hoshide et al. (7)	Intracavernous	2019	8	F	Left-sided complete CN III	STR	OZ intradural	NS	Chondroma	No	Doing well with stable CN III, IV, and VI palsies. No growth of tumor.
8	Present case	Intracavernous	2022	37	M	Diplopia, right-sided CN VI paresis	STR	Pterional	NS	Chondroma	No	Normal abducens nerve function at 1m postoperative. No growth of tumor.

CN, cranial nerve; EDA, extradural; EOR, extend of resection; FT, frontotemporal; GTR, gross total resection; NS, non-specified; OZ, orbitozygomatic; STR, subtotal resection.

### 3.2 Demographic characteristics

The seven unique cases of intracavernous chondromas identified in the literature involved patients ranging in age from 8 to 50 years (mean: 33.7 years; median: 36 years). The cohort included three males and four females, suggesting no clear sex predominance.

### 3.3 Clinical presentation

All reported cases were symptomatic at diagnosis. Clinical manifestations of CS tumors, depending on their size and extension, may include retro-orbital pain, mild exophthalmos, and impairment of cranial nerves II–VI (5, 23). In the cases reviewed here, the most frequent presentation was oculomotor nerve dysfunction. Trigeminal nerve involvement was also noted, with one patient presenting with facial numbness and another with a diminished corneal reflex.

### 3.4 Imaging features

On CT imaging, CS chondromas typically demonstrate calcifications and may show uneven or delayed contrast enhancement (19, 24). Their size and invasiveness are more readily appreciated on MRI, although no single feature reliably distinguishes them from other skull base tumors. CS chondromas are usually described as low- to isointense lesions on T1-weighted images and as having a mixed hyper- and hypointense appearance on T2-weighted images, depending on calcium content (25). Following contrast administration, enhancement is generally heterogeneous, and in some cases has been described as showing the “punica granatum seeds” sign (26).

Although rare, CS chondromas should be included in the differential diagnosis of tumors arising in the CS, including meningiomas, hemangioblastomas, schwannomas, and chondrosarcomas (17).

### 3.5 Treatment approaches

Surgical resection is the mainstay of treatment for cranial base chondromas, especially when lesions detected at an early stage (27). For symptomatic lesions, operative results have consistently been reported as superior to the natural course of the disease, but the surgical approach must be carefully tailored to each case (10, 28, 29). Intracranial chondromas are primarily extradural, and extradural techniques with middle fossa peeling—following Dolenc’s method—are generally advocated (20, 25). Among craniotomy options, the pterional approach with pretemporal extension provides sufficient exposure for chondromas located entirely within the CS (20, 29). In contrast, the orbitozygomatic (OZ) approach is best suited for lesions extending into the interpeduncular or prepontine cisterns, or involving the ipsilateral ICA or middle cerebral artery, and therefore does not offer a clear advantage for purely intracavernous tumors (30).

Removal of the sphenoid wing and the posterior part of the orbital roof, unroofing of the optic canal, and anterior clinoidectomy are usually required. The additional bony removal described in Dolenc’s technique may or may not be necessary, depending on the degree of extension of a CS chondroma.

The endoscopic endonasal approach has been described as a possible alternative, but its role remains limited for entirely intracavernous tumors because of potential lateral extension and frequent adherence to the ICA. Furthermore, this approach requires a multidisciplinary team with extensive experience to be performed safely (30, 31).

Across the seven reviewed cases, gross total resection (GTR) was achieved in four, while subtotal resection (STR) was performed in three when tumor adherence to critical neurovascular structures limited complete removal. Importantly, no STR cases showed tumor progression during follow-up, suggesting that maximal safe resection prioritizing cranial nerve preservation can also yield favorable outcomes (24).

Nonsurgical treatments such as chemotherapy or radiotherapy remain very limited for chondroma management. In cases where histopathology reveals atypia or mitotic activity and only STR is achieved, postoperative radiotherapy has been considered (25). Proton beam therapy has also been proposed as a potentially promising, though controversial, modality (29).

All included cases were symptomatic before surgical intervention, and several authors emphasized surgery as the treatment of choice in symptomatic patients (10, 21, 28).

#### 3.5.1 Craniotomy approaches and rationale for selection

Several craniotomy approaches have been reported for the management of pure intracavernous chondromas, with selection primarily driven by tumor extension, relationship to the ICA, and cranial nerve adherence. The most frequently employed approaches include OZ, frontotemporal/pterional, and pretemporal transcavernous routes.

OZ craniotomy has been preferentially used in cases with posterior or superior extension toward the interpeduncular or prepontine cisterns, providing wide exposure at the expense of more extensive bone work. In contrast, pterional or frontotemporal approaches with pretemporal transcavernous extension and extradural anterior clinoidectomy offer sufficient exposure for lesions confined entirely to the cavernous sinus, while minimizing surgical morbidity. Endoscopic endonasal approaches have been reported but remain limited for purely intracavernous tumors due to lateral extension and frequent ICA adherence. Overall, maximal safe resection rather than radical excision remains the guiding principle in approach selection.

These considerations are summarized in **Table 2**, which outlines the craniotomy approaches reported in the literature, their anatomical indications, and the rationale for extent of resection.

**Table 2:** Summary of surgical strategies and rationale across published intracavernous chondroma cases and the present case.

Case N°	Surgical approach	Route type (extradural/intradural/combined)	Tumor extension pattern	ICA/CN adherence	Extent of bony work (clinoidectomy, SOF unroofing, OZ cuts)	Extent of resection	Rationale for STR/GTR	Postoperative CN outcome
1	OZ	Extradural/intradural (NS)	Intracavernous	NS	OZ cuts	STR	Tumor adherence/limits of safe dissection	NS
2	OZ	Extradural (likely)	Intracavernous	NS	OZ cuts	GTR	Safe arachnoid planes allowed complete resection	Ophthalmoplegia 6m → resolved
3	OZ extradural	Extradural	Intracavernous	NS	OZ + extradural peeling	GTR	Reoperation for small residual enabled GTR	Transient VI palsy
4	Pterional	Extradural/interdural	Intracavernous/interdural	Likely CN adherence	Pterional bone work	STR	Adherence to CNs limited safe removal	Pain resolved; diplopia improved POD1
5	FT + EDA	Extradural	Intracavernous	NS	Clinoidectomy likely	GTR	Favorable anatomy permitted full removal	NS
6	FT	Extradural	Intracavernous	NS	FT approach bone work	GTR	Accessible planes; complete resection achieved	Normal CN VI POD1
7	OZ intradural	Intradural	Intracavernous	CN adherence	OZ cuts	STR	Adherence to multiple CNs	Stable III/IV/VI; no growth
8	Pterional	Extradural	Intracavernous	ICA adherence	Clinoidectomy + SOF unroof	STR	Residual calcified fragment adherent to ICA left	Normal CN VI at 1m; stable

CN, cranial nerve; EDA, extradural anterior clinoidectomy; EOR, extent of resection; FT, frontotemporal; GTR, gross total resection; ICA, internal carotid artery; NS, not specified; OZ, orbitozygomatic; POD, postoperative day; SOF, superior orbital fissure; STR, subtotal resection.

### 3.6 Histopathological findings

Definitive diagnosis of CS chondroma is based on anatomopathological findings. Histologically, these tumors are typically composed of mature hyaline cartilage (1). Malignant sarcomatous transformation has been reported, particularly in association with syndromes of multiple enchondromatosis (32). Yasargil described a case initially diagnosed as a chondroma, which was later reclassified as a chondrosarcoma; the discrepancy was attributed to sampling from a benign area within the chondrosarcoma (3). These tumors lack distinctive pathognomonic features, and the histopathologic differential diagnosis includes osteochondroma and well-differentiated chondrosarcoma. Immunohistochemically, chondromas are often positive for S-100 and vimentin, but negative for epithelial membrane antigen and, in typical cases, Ki-67. In contrast, chondrosarcomas show increased cellularity, nuclear atypia, mitotic activity, and Ki-67 positivity (1, 15).

### 3.7 Outcomes

Radical but safe resection remains the preferred strategy; however, given the slow growth of chondromas, even STR can yield durable control and favorable outcomes (25). Neurological deficits often improve postoperatively, with cranial nerve recovery typically occurring within the first year. In particular, oculomotor palsy has been reported to improve within the first 6 months (5, 21).

## 4 Discussion

Our literature review identified seven cases of CS chondromas primarily originating within the sinus (**Table 1**) (4, 5, 7, 8, 17–20). The mean age was 33.4 years (range, 8–50 years), with a slight female predominance (4 women, 3 men). Cranial neuropathies were the most frequent presenting symptoms, with oculomotor nerve impairment being the most common manifestation. GTR was achieved in 57% (4/7) of cases, including one that required reoperation to achieve complete removal. OZ craniotomy was the most frequently used approach (57%, 4/7). Histopathology consistently showed mature hyaline cartilage without atypia or mitotic activity. None of the reported patients received adjuvant radiotherapy, radiosurgery, or chemotherapy. Follow-up was available for 5 of the 7 cases, and no recurrences, new postoperative cranial nerve deficits, or malignant transformations were documented, supporting the efficacy of surgical resection in disease control.

Our present case—a 37-year-old male with progressive diplopia due to a CS chondroma—aligns with the demographic profile reported in the literature, where most patients presented in early to middle adulthood with cranial neuropathies, particularly oculomotor involvement. Unlike the majority of reviewed cases that utilized an OZ approach, we achieved near-total resection via a pterional craniotomy with pretemporal transcavernous extension and extradural anterior clinoidectomy. Similar to previously reported STR, a small residual calcified fragment was intentionally left adherent to the ICA to preserve neurovascular integrity. Postoperatively, the patient experienced complete resolution of diplopia without new deficits, and follow-up imaging at one and two months confirmed stability of the residual lesion. These findings reinforce the literature trend that maximal safe resection—rather than radical removal at the expense of cranial nerve function—provides favorable functional outcomes with durable disease control. These observations, summarized alongside previously published cases in **Table 2**, further highlight how our operative strategy and outcomes align with established trends in the management of purely intracavernous chondromas.

### 4.1 Surgical considerations

Surgical approaches to the CS remains one of the most technically demanding areas due to its dense neurovascular anatomy. Approaches to this region gained prominence in the mid-1980s with Dolenc’s intra- and extradural techniques (6, 20), while further refinements were pioneered by Browder (33), Parkinson (6), and Hakuba (28). In the reviewed cases, microsurgical approaches—most commonly OZ and pterional craniotomies—enabled safe tumor removal. Complete resection is desirable; however, safe STR should be prioritized if tumor adherence to critical neurovascular structures is encountered. Our case exemplifies this principle, as deliberate preservation of a calcified fragment attached to the ICA avoided morbidity without compromising early outcomes.

The prognosis of CS chondromas appears favorable. In our review, no cases with available follow-up demonstrated recurrence or malignant transformation, even after STR. Cranial neuropathies frequently improved within months after surgery, particularly oculomotor deficits. These findings underscore the importance of long-term clinical and radiological surveillance, given the slow-growing nature of chondromas and the potential—albeit rare—for malignant progression reported in other intracranial locations.

### 4.2 Limitations of the literature

The available evidence is limited to single case reports and small case series, with heterogeneous surgical approaches and variable follow-u This restricts the ability to draw robust conclusions about optimal management strategies or long-term outcomes. Furthermore, reporting of immunohistochemical markers and follow-up data is inconsistent across publications.

## 5 Conclusion

CS chondroma remains a rare entity, and its resection continues to pose a neurosurgical challenge. Although the precise etiology is not fully understood, several theories regarding its origin have been proposed. Our review of the literature demonstrates that surgical resection is the mainstay of treatment, particularly in symptomatic patients, as it consistently results in improvement of neurological impairment. Extradural approaches to the CS are often recommended to optimize surgical exposure and safety. Selection of the craniotomy approach should be individualized, with pterional or pretemporal transcavernous routes being sufficient for purely intracavernous lesions, while more extensive approaches are reserved for tumors with posterior or cisternal extension. Given the slow growth of these tumors, even STR can provide durable disease control and favorable outcomes. When histopathological evaluation reveals atypia or mitotic activity and only STR is feasible, postoperative adjuvant treatments—such as radiotherapy or proton beam therapy—may be considered.

## Data Availability

The datasets presented in this article are not readily available because of ethical and privacy restrictions. Requests to access the datasets should be directed to the corresponding author/s.
